# Evaluation of response to neoadjuvant chemotherapy in osteosarcoma using dynamic contrast-enhanced MRI: development and external validation of a model

**DOI:** 10.1007/s00256-023-04402-8

**Published:** 2023-07-18

**Authors:** Gijsbert M. Kalisvaart, Thomas Van Den Berghe, Willem Grootjans, Maryse Lejoly, Wouter C. J. Huysse, Judith V. M. G. Bovée, David Creytens, Hans Gelderblom, Frank M. Speetjens, Lore Lapeire, Michiel A. J. van de Sande, Gwen Sys, Lioe-Fee de Geus-Oei, Koenraad L. Verstraete, Johan L. Bloem

**Affiliations:** 1 Department of Radiology and Nuclear Medicine, Leiden University Medical Center, Albinusdreef 2, 2333 ZA Leiden, The Netherlands; 2 Department of Radiology and Medical Imaging, Ghent University Hospital, Ghent, Belgium; 3 Department of Pathology, Leiden University Medical Center, Albinusdreef 2, 2333 ZA Leiden, The Netherlands; 4 Department of Pathology, Ghent University Hospital, Ghent, Belgium; 5 Department of Medical Oncology, Leiden University Medical Center, Albinusdreef 2, 2333 ZA Leiden, The Netherlands; 6 Department of Medical Oncology, Ghent University Hospital, Ghent, Belgium; 7 Department of Orthopedics, Leiden University Medical Center, Albinusdreef 2, 2333 ZA Leiden, The Netherlands; 8 Department of Orthopedics, Ghent University Hospital, Ghent, Belgium

**Keywords:** Osteosarcoma, Response monitoring, Neoadjuvant chemotherapy, Dynamic contrast-enhanced MRI, Histological response, External validation

## Abstract

**Objective:**

To identify which dynamic contrast-enhanced (DCE-)MRI features best predict histological response to neoadjuvant chemotherapy in patients with an osteosarcoma.

**Methods:**

Patients with osteosarcoma who underwent DCE-MRI before and after neoadjuvant chemotherapy prior to resection were retrospectively included at two different centers. Data from the center with the larger cohort (training cohort) was used to identify which method for region-of-interest selection (whole slab or focal area method) and which change in DCE-MRI features (time to enhancement, wash-in rate, maximum relative enhancement and area under the curve) gave the most accurate prediction of histological response. Models were created using logistic regression and cross-validated. The most accurate model was then externally validated using data from the other center (test cohort).

**Results:**

Fifty-five (27 poor response) and 30 (19 poor response) patients were included in training and test cohorts, respectively. Intraclass correlation coefficient of relative DCE-MRI features ranged 0.81–0.97 with the whole slab and 0.57–0.85 with the focal area segmentation method. Poor histological response was best predicted with the whole slab segmentation method using a single feature threshold, relative wash-in rate <2.3. Mean accuracy was 0.85 (95%CI: 0.75–0.95), and area under the receiver operating characteristic curve (AUC-index) was 0.93 (95%CI: 0.86–1.00). In external validation, accuracy and AUC-index were 0.80 and 0.80.

**Conclusion:**

In this study, a relative wash-in rate of <2.3 determined with the whole slab segmentation method predicted histological response to neoadjuvant chemotherapy in osteosarcoma. Consistent performance was observed in an external test cohort.

**Supplementary Information:**

The online version contains supplementary material available at 10.1007/s00256-023-04402-8.

## Introduction

Presence of residual viable osteosarcoma tissue following neoadjuvant chemotherapy is a prognostic factor [1]. Although histological determination of percentage viable tumor on the resected specimen has limitations, it is still the reference standard for response [1, 2]. An imaging method allowing prediction of this histological response before resection could therefore provide tools for personalization of (neo)adjuvant chemotherapy. In this regard, several imaging techniques including dynamic contrast-enhanced (DCE-)MRI, diffusion-weighted MRI and [^18^F]FDG PET-CT have been proposed [3–6]. Of particular interest is DCE-MRI, which has been shown to allow identification of viable tumor compartments in osteosarcoma after chemotherapy [3, 7–10]. As gadolinium-enhanced MRI is included in the standard protocol for tumor imaging, DCE-MRI is the only of these methods that does not have a time penalty [11]. Although results look promising, there is currently no guideline nor consensus on how to use DCE-MRI for response characterization in osteosarcoma patients [11, 12]. Kubo et al. concluded in a meta-analysis that features of the time-intensity curve derived from DCE-MRI can be useful in predicting histological response, but that a significant heterogeneity in prediction performance exists. Small patient populations, heterogeneous methods, limited statistical power and lack of external validations were limitations to the included studies [10].

In this study, we investigated DCE-MRI features in a relatively large population and validated performance for predicting histological response in an external cohort. Two frequently used methods for region-of-interest (ROI) selection (whole slab and focal area method) and changes in DCE-MRI features during chemotherapy were used to create models. Furthermore, the added value of DCE-MRI features to changes in tumor volume for histological response prediction was assessed.

## Methods

### Patient inclusion

Approval by the institutional review boards was obtained prior to the study, and the need for written informed consent was waived due to the retrospective character of the study (protocols B19.050/BC-09111). Histologically confirmed osteosarcoma patients who underwent MRI pre- and post-neoadjuvant chemotherapy and subsequent resection between 2005 and 2020 in either Leiden University Medical Center or Ghent University Hospital were retrospectively included in a training and test cohort (for external validation), respectively. Exclusion criteria were MRI performed in different centers (*n*=165), DCE-MRI not performed pre- and post-neoadjuvant chemotherapy (*n*=194), usage of non-identical DCE-MRI scan protocols pre- and post-neoadjuvant chemotherapy (*n*=50) and history of previous surgery, radiotherapy or chemotherapy (*n*=7). Therefore, out of 501 initially enrolled patients, a total of 416 were excluded, leaving 85 patients eligible for analysis. Patient age, gender, tumor location, subtype, timing of imaging relative to treatment, neoadjuvant treatment and histological response were documented to assess patient characteristics in the training and test cohort (Supplementary Material 1).

### MRI protocol

Protocol details for both centers are provided in Supplementary Material 2. DCE-MRI series of the entire tumor volume were acquired of all patients in the training cohort and in 37% of patients in the test cohort. Of the remaining 63% of patients in the test cohort, acquired DCE-MRI series consisted of a single slice of the largest tumor area as determined on previous non-dynamic images. Acquisition of DCE-MRI series was started 6 s before intravenous injection of 0.2ml/kg gadolinium contrast medium (0.5mmol/ml) with 2ml/s by means of an automatic injector. Temporal resolution during the first minute was 1 s, and from minute 2 to 5, 3–4 s. Subtraction images of the DCE-MRI sequence in which the first baseline image is subtracted from all subsequent images were generated automatically to support visual detection of early and fast enhancing regions and allow calculation of DCE-MRI features.

### Imaging assessment and imaging features

All DCE-MRI images were processed in Philips Intellispace (version 10.1, Philips Medical Systems Nederland B.V., Best, The Netherlands). All images were independently segmented by G.M.K. (4 years of experience) and T.V.D.B. (4 years of experience) under supervision of two experienced musculoskeletal radiologists, J.L.B. (34 years of experience) and K.L.V. (33 years of experience), respectively, for assessment of interobserver variability. Measurement of largest tumoral diameters was performed on conventional T1- or fat-saturated T2-weighted static images. Segmentations and measurements were performed manually, blinded for histological and clinical information.

**Fig. 1:**
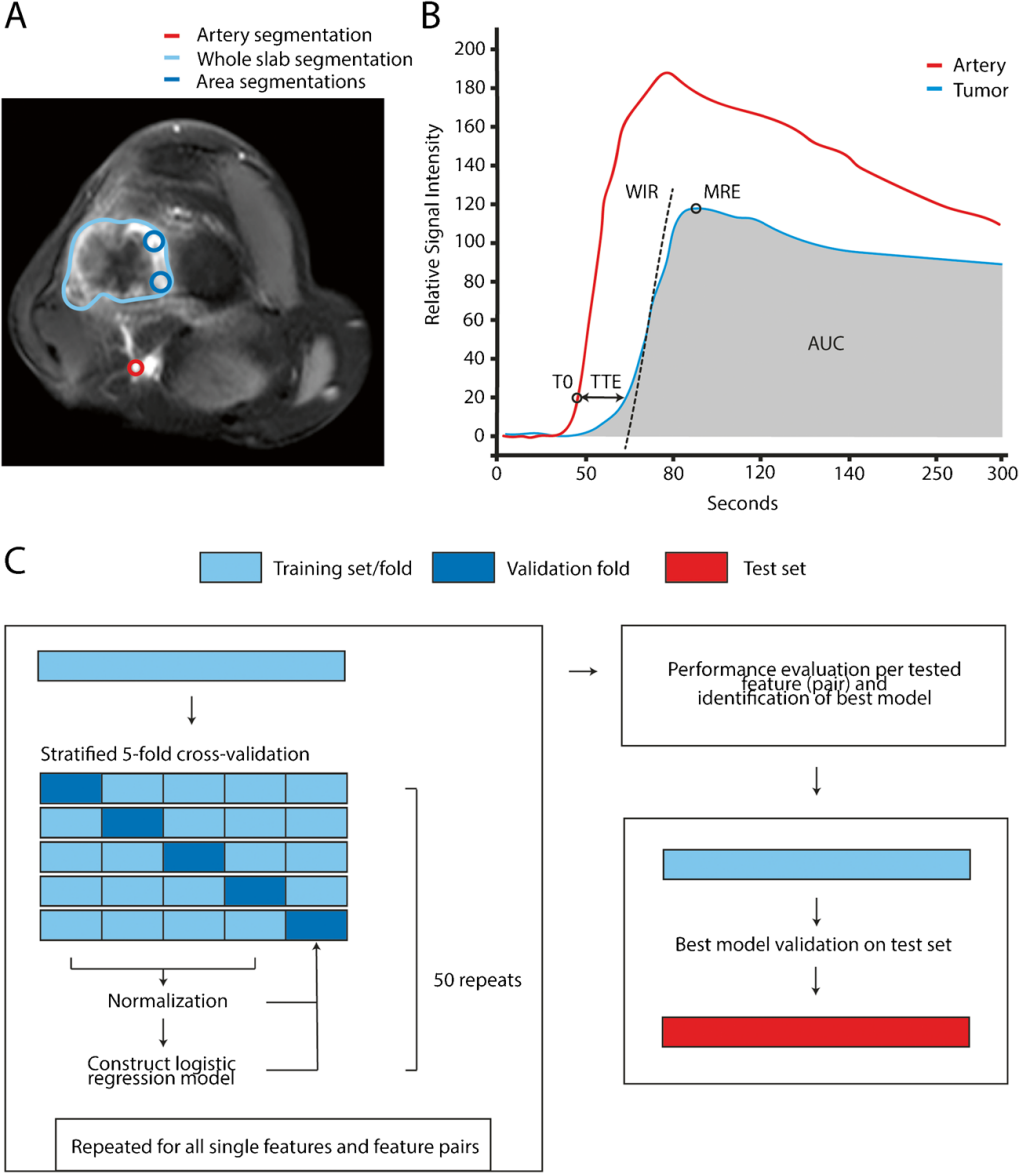
Overview of segmentation methods, feature extraction, model building and model testing. **A**; Dynamic contrast-enhanced MRI image of an osteosarcoma in the distal femur showing the 2D segmentation of the entire tumor (whole slab, light blue area) and regions-of-interest placed in the most intensely and early enhancing parts of the tumor (focal area method, dark blue circles). Arterial segmentation (red circle) is performed as reference tissue comparison to the tumoral segmentations. **B**; Schematic dynamic contrast-enhanced MRI derived time-intensity curve and perfusion features for an artery and a tumor region depicting changes in average pixel signal intensity over time due to influx and outflux of gadolinium contrast medium and its distribution over the vascular versus tumoral compartments. The features time to enhancement (TTE), wash-in rate (WIR), maximum relative enhancement (MRE) and area under the curve (AUC) are illustrated. **C**; Overview of model training with logistic regression, internal cross-validation, model selection and external validation on a test cohort. Models were cross-validated to test all single features separately and all feature pairs per segmentation method. AUC area under the curve, MRE maximum relative enhancement, T0 time of onset of enhancement, TTE time to enhancement, WIR wash-in rate

Two methods were used to segment ROIs, corresponding to the methods used in previous studies [3, 7–10]. For the whole slab method, the entire tumor was segmented on a single slice containing the largest tumor area. For the focal area method, two circular ROIs of approximately 10–20mm^2^ were placed within the earliest and fastest enhancing regions of the tumor, as identified on the subtracted DCE-MRI images (Fig. 1A). When the start of enhancement between areas could not be differentiated, two areas with visually most intense enhancement were segmented. In addition, a circular ROI was placed into the artery closest to the tumor. From the ROIs, time-intensity curves (TICs) were created. A total of four perfusion features on both the pre- and post-neoadjuvant chemotherapy DCE-MRI images were derived from the TICs (Fig. 1B). These features were selected based on previous studies and the ability to capture different aspects of TICs [13, 14]. For calculation of these features, the time of onset of enhancement (T0) had to be determined for all ROIs of the tumor and closest regional artery. T0 is defined as the first time point after starting the DCE-MRI acquisition at which the signal intensity increases for more than 20% compared to the mean signal intensity at baseline (all points in time before T0). Subsequently, the time to enhancement (TTE), wash-in rate (WIR), maximum relative enhancement (MRE) and area under the curve (AUC) were determined. TTE is defined as the time difference between T0 in the tumor and the regional artery. WIR is defined as the maximum rise in signal intensity per second between T0 of the tumor and the time point of maximum enhancement (maximum signal intensity) relative to the signal intensity on the non-enhanced baseline image. MRE is defined as the maximum signal intensity relative to the signal intensity on non-enhanced images. AUC is defined as the integral of the signal intensities over time relative to the signal intensity at baseline. For the focal area method, the DCE-MRI features from the two ROIs were averaged for further analyses. In order to capture changes in tumor biology over time, relative change of WIR, MRE and AUC (rWIR, rMRE and rAUC, respectively) was calculated by dividing the features on pre-neoadjuvant chemotherapy by post-neoadjuvant chemotherapy images. Furthermore, the difference in TTE was determined by subtracting pre- from post-neoadjuvant chemotherapy TTE (ΔTTE). Finally, changes in tumor volume were determined by dividing tumor volume (estimated as the volume bounded by an ellipsoid based on tumor diameters) on pre-neoadjuvant chemotherapy images by the tumor volume on the post-neoadjuvant chemotherapy images (rVolume).

### Reference standard—histological response assessment

Histological response was determined on a complete coronal, sagittal or transverse slab of the resected tumor through its largest dimension according to current guidelines for histological response assessment [12, 15–17]. Good histological response was defined as <10% viable tumor cells and poor histological response as ≥10% viable tumor after neoadjuvant chemotherapy [1, 2]. In the training cohort, histological response was extracted from the patient files and reassessed and rescored by an expert pathologist (J.V.M.G.B., 25 years of experience) in border-line cases to optimize the reference standard. Due to difference in scoring methods in the past, response was rescored blinded for pathological reports for all patients from the test cohort by an expert pathologist (D.C., 15 years of experience) to assure consistency. Both in the training and test cohort, the pathologist was fully blinded for clinical and imaging features. On average, the time interval between DCE-MRI evaluation and resection was 18 and 8 days in the training and test cohort, respectively.

### Statistical analysis

The intraclass correlation coefficient (ICC) was used for assessment of interobserver agreement for all relative imaging features and tumor volume in the training cohort. Differences in features between good response and poor response in the training cohort were compared using the Kruskall-Wallis test. Significance level (*p*-value) was set at 0.05. Before modeling histological response, all features were log transformed to reduce the effects of data skewness. In the training cohort, cross-validation was performed (5-folds, 50 repeats) to build models and evaluate performance of the different features (and feature combinations) (Fig. 1C). Within every training and validation fold combination, data was normalized on training fold data. Modeling was performed using logistic regression. This model cross-validation was performed once with all single features separately, once with all seperate perfusion features combined with rVolume, and once with all combinations of two perfusion features within a segmentation method. Due to the limited cohort size, the maximum number of included features was limited to two. The mean area under the receiver operating characteristic curve (AUC-index) was used to determine the model with the best performance for poor versus good response prediction. The 95% confidence intervals (95%CI) for performance scores were constructed using corrected resampled *t*-test [18]. The optimal probability threshold in the training cohort was determined with the Youden’s index [19]. The best performing model and corresponding probability threshold were tested twice in the external test cohort, once with, and once without using the combating batch effects while combining batches (ComBat) method to harmonize test cohort data to training cohort data. The aim of this harmonization step is to reduce center specific effects, such as differences in timing of post-neoadjuvant chemotherapy DCE-MRI (typically performed on the last day of neoadjuvant chemotherapy in the training cohort and on average 9 days later in the test cohort) while attempting to preserve biological meaning in the data [20–22]. Statistical analysis was performed in Python (Python Software Foundation, Python Language Reference, version 3.7. Available at http://www.python.org).

## Results

### Study sample and patient characteristics

A total of 55 patients (median age 20) were included in the training cohort and 30 (median age 15) were included in the test cohort. In the training cohort, 27 (49%) patients had a poor response to neoadjuvant chemotherapy. In the test cohort, 19 (63%) patients had a poor response. Patient characteristics are shown in Supplementary Material 1.

### Interobserver variability and feature analysis

**Fig. 2:**
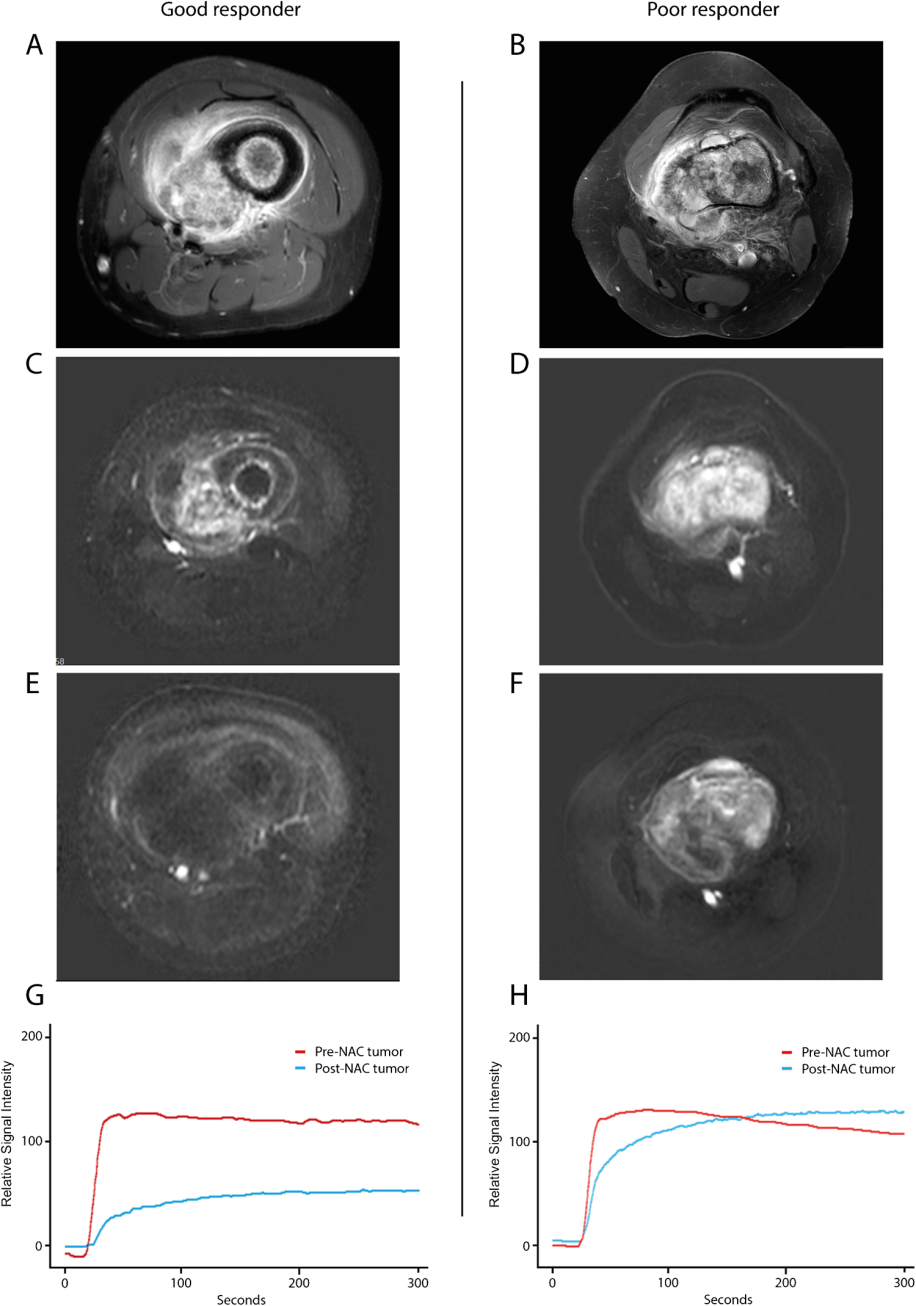
Differences in (dynamic) contrast-enhanced MRI images and time-intensity curve analysis in osteosarcoma patients between good and poor responders to neoadjuvant chemotherapy. **A–B**; Pre-neoadjuvant chemotherapy T1-weighted gadolinium contrast-enhanced spectral pre-saturation with inversion recovery (SPIR) images of an osteosarcoma in the femur diaphysis and in the distal femur in a good (**A**) and poor (**B**) responder, respectively. **C–D**; Subtraction images of the pre-neoadjuvant chemotherapy dynamic contrast-enhanced MRI sequence at 6 s after arrival of gadolinium contrast medium in a good (**C**) and poor (**D**) responder. **E–F**; Subtraction images of the post-neoadjuvant chemotherapy dynamic contrast-enhanced MRI sequence at 6 s after arrival of gadolinium contrast medium showing an absence of residual enhancement in the good responder (**E**) and persistent and heterogeneous enhancement of the poor responder (**F**). After resection, the percentage of viable tumor cells post-neoadjuvant chemotherapy was estimated to be 1% versus 20–40% in the good and poor responder, respectively. **G–H**; Dynamic contrast-enhanced MRI-derived time-intensity curves in corresponding tumors (whole slab method) pre-neoadjuvant chemotherapy (red) and post-neoadjuvant chemotherapy (blue) in a good responder (**G**) and in a poor responder (**H**). NAC neoadjuvant chemotherapy

**Fig. 3:**
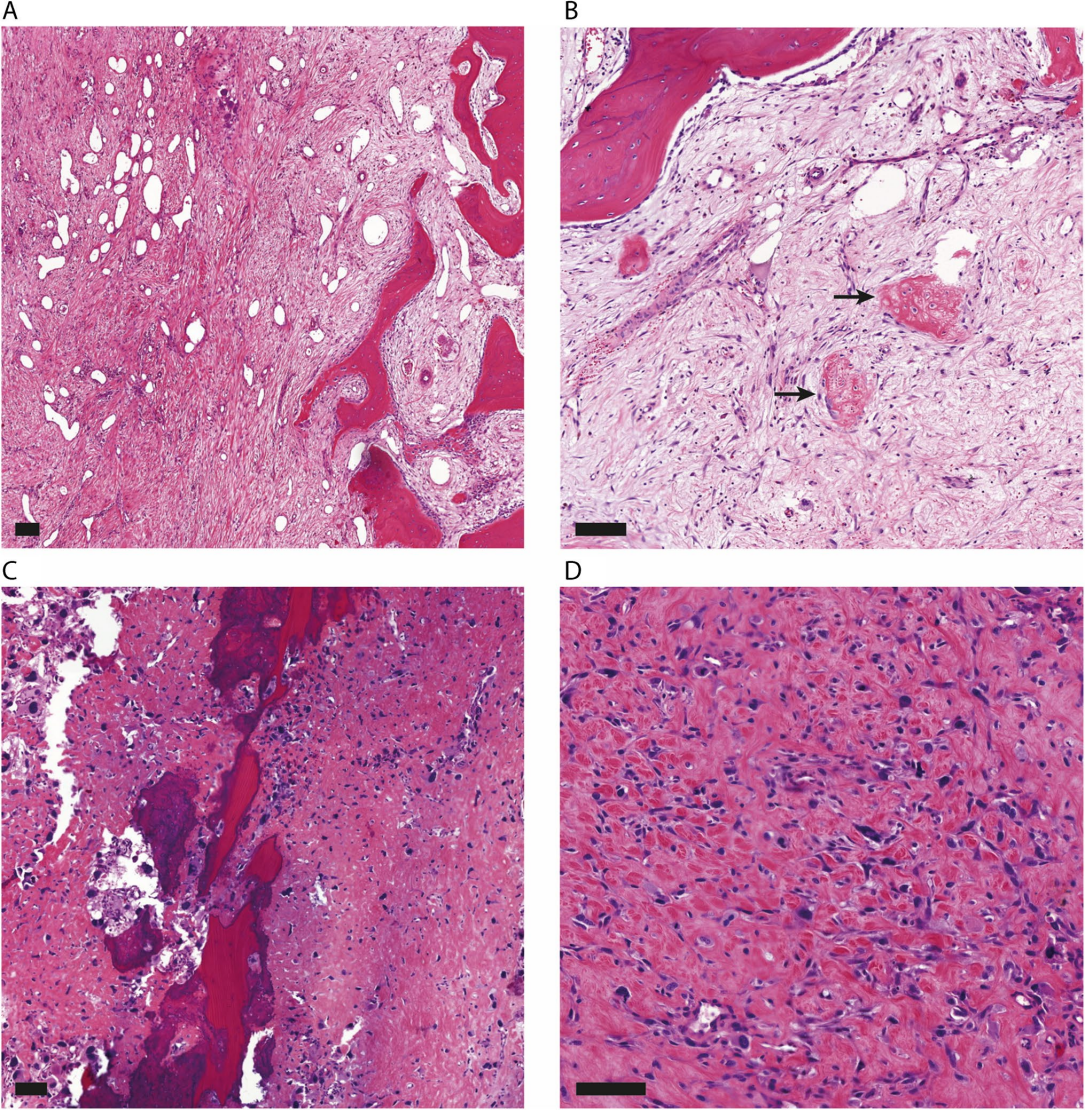
Representative histology of osteosarcomas depicted in Fig. 2 (good versus poor responder). Black scale bars represent 100 μm. **A–B**; Good responder post-neoadjuvant chemotherapy with 1% vital tumor cells remaining and showing loose edematous as well as more condensed fibrotic areas with remnants of tumor osteoid (arrows). **C–D**; Poor responder post-neoadjuvant chemotherapy with 30% vital tumor cells remaining and showing pre-existing lamellar bone, surrounded by a proliferation of pleomorphic and vital tumor cells depositing osteoid

The range of ICC values for the relative DCE-MRI features was 0.81–0.97 for the whole slab and 0.57–0.85 for the focal area method (Table 1). ICC for relative change in tumor volume was 0.72. Relative feature values were significantly lower in the poor response compared to the good response group for all features (*p*-values <0.001) (Table 1). Figures 2 and 3 show examples of (DCE-)MRI and histology in a good and a poor responder to neoadjuvant chemotherapy.

**Table 1:** Intraclass correlation coefficients with 95% confidence intervals for predictive features and feature summaries expressed as median and interquartile range in the training cohort with comparison of the good versus poor responder groups. *p*-values represent statistical differences in feature values between good and poor responders calculated with the Kruskall-Wallis test

	ICC and 95% CI	Feature values (median and interquartile range)			***p***-**value**
		Training cohort (*n*=55)	Good responders (*n*=28)	Poor responders (*n*=27)	
rVolume	0.72 (0.43–0.86)	0.97 (0.71–1.76)	1.43 (0.95–2.02)	0.78 (0.64–1.00)	< 0.001
Whole slab method					
rWIR	0.81 (0.68–0.89)	1.92 (0.69–5.67)	4.81 (2.52–13.45)	0.69 (0.43–1.14)	< 0.001
rMRE	0.95 (0.91–0.97)	1.35 (0.85–2.97)	2.66 (1.46–7.68)	0.85 (0.72–1.33)	< 0.001
rAUC	0.97 (0.94–0.98)	1.65 (0.72–5.81)	3.90 (1.87–16.23)	0.72 (0.46–1.27)	< 0.001
ΔTTE	0.85 (0.60–0.91)	1.54 (–0.01–6.15)	7.04 (3.47–24.76)	0.00 (–3.08–3.09)	< 0.001
Focal area method					
rWIR	0.81 (0.67–0.89)	1.47 (0.63–3.67)	3.22 (1.93–7.53)	0.63 (0.46–1.21)	< 0.001
rMRE	0.58 (0.28–0.76)	1.38 (0.83–1.99)	1.86 (1.42–2.59)	0.83 (0.60–1.36)	< 0.001
rAUC	0.85 (0.74–0.91)	1.19 (0.65–2.34)	2.16 (1.20–4.28)	0.65 (0.52–0.82)	< 0.001
ΔTTE	0.57 (0.25–0.75)	0.93 (0.80–1.25)	4.68 (–1.70–10.31)	0.00 (–1.96–1.54)	< 0.001

95%CI 95% confidence interval, ΔTTE delta time to enhancement, ICC intraclass correlation coefficient, n number, p probability, rAUC relative area under the curve, rMRE relative maximum relative enhancement, rVolume relative volume, rWIR relative wash-in rate

### Imaging assessment, imaging features, model performance and selection

**Fig. 4:**
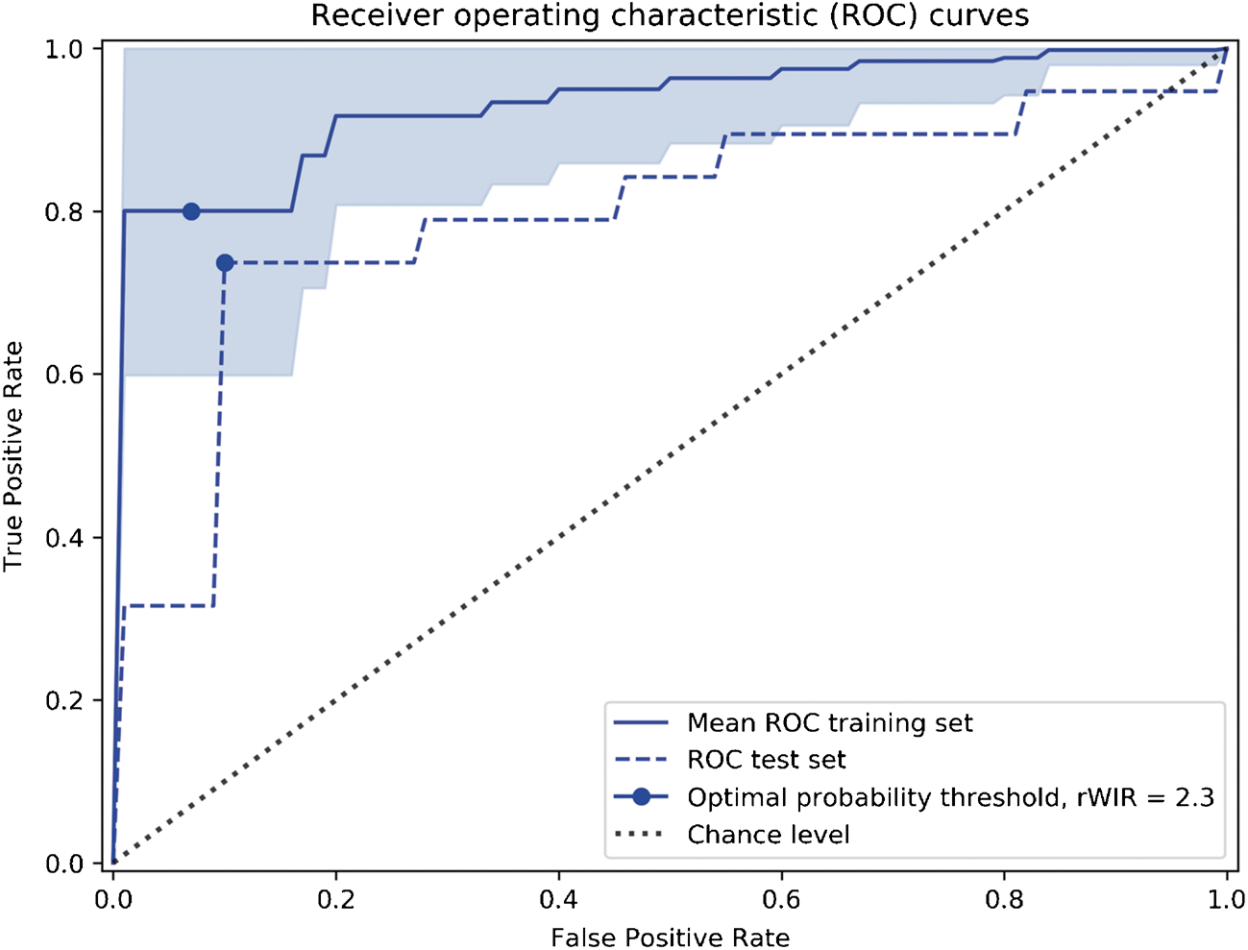
Receiver operating characteristic curves for the final model (rWIR as a single feature of the whole slab segmentation method) with internal cross-validation and external validation on the test cohort results. ROC receiver operating characteristic, rWIR relative wash-in rate

Modeling solely based on tumor volume had, in cross-validation, a mean accuracy of 0.70 (95%CI: 0.59–0.82) and AUC-index of 0.75 (95%CI: 0.61–0.90). Mean AUC-index ranges in cross-validations were 0.84–0.93 for the whole slab and 0.83–0.93 for the focal area method (Table 2). The best performing segmentation method and modeling combination was whole slab method-based and included only rWIR. The cross-validated means for this model were 0.85 (95%CI: 0.75–0.95) for accuracy, 0.90 (95%CI: 0.76–1.00) for sensitivity, 0.81 (95%CI: 0.63–0.99) for specificity and 0.93 (95%CI: 0.86–1.00) for AUC-index. Because the best performing model included only 1 feature, a threshold for prediction of poor histological response of rWIR <2.3 was determined (Fig. 4).

**Table 2:** Area under the receiver operating characteristic curve (AUC-index) with 95% confidence intervals in cross-validation of the training cohort for all predictive features separately and combined in pairs per segmentation method

Features		Single feature	Paired with			
			rMRE	rAUC	ΔTTE	rVolume
Whole slab method	rWIR	0.93 (0.86–1.00)^a^	0.92 (0.84–1.00)	0.93 (0.85–1.00)	0.93 (0.85–1.00)	0.93 (0.85–1.00)
	rMRE	0.86 (0.74–0.97)	-	0.90 (0.82–0.99)	0.86 (0.75–0.97)	0.84 (0.72–0.97)
	rAUC	0.89 (0.80–0.97)	-	-	0.93 (0.86–0.99)	0.90 (0.82–0.99)
	ΔTTE	0.88 (0.78–0.98)	-	-	-	0.85 (0.73–0.97)
Focal area method	rWIR	0.92 (0.84–1.00)	0.93 (0.85–1.00)	0.92 (0.84–1.00)	0.93 (0.86–0.99)	0.91 (0.82–0.99)
	rMRE	0.88 (0.77–0.99)	-	0.84 (0.73–0.94)	0.83 (0.71–0.95)	0.88 (0.77–0.99)
	rAUC	0.89 (0.79–0.98)	-	-	0.89 (0.79–0.98)	0.89 (0.80–0.98)
	ΔTTE	0.83 (0.71–0.96)	-	-	-	0.83 (0.71–0.95)
rVolume		0.75 (0.61–0.90)	–	-	-	-

ΔTTE delta time to enhancement, rAUC relative area under the curve, rMRE relative maximum relative enhancement, rVolume relative volume, rWIR relative wash-in rate

^a^The highest mean area under the receiver operating characteristic curve and 95% confidence interval was observed when testing rWIR, determined with whole slab segmentation, as a single feature (not combined with another feature in a pair)

### External validation on a test cohort

In external validation after ComBat harmonization in an unseen test cohort, this model showed an accuracy of 0.80, sensitivity of 0.74, specificity of 0.91 and AUC-index of 0.80. External validation without the use of Combat harmonization resulted in an accuracy of 0.70, sensitivity of 0.58, specificity of 0.91 and AUC-index of 0.80.

## Discussion

In a relatively large patient group, internal and external validation showed that changes in DCE-MRI features between start and completion of neoadjuvant chemotherapy are associated with histological response in patients with osteosarcoma. The best performing model included only rWIR derived from the whole slab segmentation method with an optimal threshold of <2.3 for prediction of poor response. The use of one feature based on segmentation of the largest tumor slab rather than subjectively selecting ROIs of the highest enhancing regions makes this method straightforward to implement. The model showed good classification performance to distinguish poor from good histological response before surgical resection.

The use of DCE-MRI in predicting tumor response is not new. However, due to limited cohort sizes, cohort heterogeneity and absence of external validation, studies on DCE-MRI or other imaging modalities have not yet resulted in a widely accepted method for response assessment. Reported accuracies generally range between 0.60 and 0.77 [3, 5, 6, 23]. The determined threshold for prediction of poor response of rWIR <2.3, corresponding to a <57% decrease in WIR over time, is of the same order of magnitude as the threshold of <60% decrease in the WIR studied in the meta-analysis by Kubo et al., with a reported pooled sensitivity, specificity and AUC-index of 0.73, 0.83 and 0.89, affirming the robustness of the current results [10].

Decrease in performance between internal cross-validation in the training cohort and external validation reported in the current study is partially explained by differences in MRI vendor, scan protocols and cohort characteristics between centers. For example, in 63% of 30 scans in the external validation test cohort, DCE-MRI images were acquired in one slice, making it impossible to assess the entire tumor on the dynamic images. Furthermore, median days between last neoadjuvant chemotherapy and response scans was 9 days longer in the test cohort as compared to the training cohort. As expected, accuracy was lower when ComBat was not used to correct for these center specific effects.

DCE-MRI reflects tumoral vascularization, perfusion and capillary permeability, which are hallmarks of tumor viability [24]. This allows differentiation between remaining viable osteosarcoma cells and histological reaction to chemotherapy such as necrosis, fibrosis, oedema and hemorrhage (Figs. 2 and 3) [8, 9]. The current results show significantly larger decrease in WIR, MRE and AUC and increase in TTE in good responders in comparison to poor histological responders for both the focal area and whole slab segmentation method. Heterogeneity of osteosarcoma has been the rationale for using the focal area segmentation method. Viable tumor parts show relatively early, fast and intense enhancement compared to less viable tumor parts [25]. However, whole slab segmentation provides an overall estimation of average tumor response in the largest slice of the tumor on cross section, which is more similar to the histological response assessment as proposed by the World Health Organization [16]. Interobserver variability for the whole slab segmentation method was lower than for the focal area segmentation method. This is expected because the focal area method requires identification of the earliest and fastest enhancing tumor areas on the subtraction images, a process that is subjective. In this regard, predictive performance and repeatability might be improved by performing 3D and automated tumor segmentations to make volume estimations more precise in future studies.

Modeling DCE-MRI features outperformed modeling solely based on changes in tumor volume. While the intra-osseous tumor volume does not change, the soft tissue component may decrease in size following neoadjuvant therapy. However, there is consensus in literature that change in tumor volume is unreliable for response monitoring in osteosarcomas [26–28]. Although in our study good responders typically exhibited more tumor shrinkage, change in tumor volume was less predictive than changes in perfusion characteristics and did not add any predictive value to our models.

The chosen focus of our study on DCE-MRI has several limitations. The number of excluded patients is large because many patients were referred to our centers with MR studies that did not have adequate DCE-MRI protocols. The retrospective nature of our study did not give us the opportunity to repeat these MR studies. Also in our clinical DCE-MRI protocol we did not add T1-mapping, which excludes pharmacokinetic modeling of quantitative permeability parameters as an option [3, 29]. A combination of DCE-MRI with [^18^F]FDG-PET and diffusion-weighted imaging, quantifying glucose metabolism and cellularity respectively, might further improve understanding of biological changes in osteosarcomas during therapy [3, 5, 6, 23, 30]. Another limitation of the current study is the subjective method to determine percentage of viable tumor in histology and the absence of direct matching of imaging and histopathological slabs. This method is still considered the optimal reference standard as it has been shown to correlate with prognosis. However, more recent studies report conflicting results on its association with survival [1, 31, 32]. Future research should therefore aim to test predictive imaging features for histological response and especially survival prediction.

Although response evaluation is currently not used to modulate neoadjuvant therapy, a method that can accurately determine or predict response at an individual patient level may have this potential. In this regard, the challenge of establishing the level of accuracy needed for such a method to be of clinical value should be acknowledged. Ultimately, accurate identification prior to treatment of patients with osteosarcoma who will benefit from neoadjuvant chemotherapy might potentially have an impact on treatment strategies. Quantification of tumor heterogeneity on pre-treatment imaging using a radiomic texture analysis on T1-weighted MRI, CT and [^18^F]FDG-PET data before neoadjuvant chemotherapy has been used to predict response to neoadjuvant therapy in patients with osteosarcoma [33–35].

In conclusion, monitoring perfusion characteristics based on DCE-MRI at diagnosis and after neoadjuvant chemotherapy is predictive for histological response in osteosarcoma. The proposed model shows a good discrimination of poor and good histological response to neoadjuvant chemotherapy in an external validation test cohort. The model only includes the relative wash-in rate, providing a threshold for response evaluation of 2.3. This feature is thus one of the important biomarkers that can be used in future multimodality studies.

### Supplementary information

ESM 1 (17.4 KB)

ESM 2 (13.8 KB)

## Data Availability

Data generated or analyzed during the study are available upon reasonable request.
