# Supplementary material for: Evaluation of response to neoadjuvant chemotherapy in osteosarcoma using dynamic contrast-enhanced MRI: development and external validation of a model
Source: Skeletal Radiol. 2023 Jul 18;53(2):319–28. doi: 10.1007/s00256-023-04402-8 (PMC10730632; doi:10.1007/s00256-023-04402-8)
Supplement: Supplementary file 1 — ESM 1 (17.4 KB) [file 256_2023_4402_MOESM1_ESM.docx]

**Supplementary Material 1**

|  | Training cohort (n=55) | | | Test cohort (n=30) | | |
| --- | --- | --- | --- | --- | --- | --- |
|  | **Good responders  (n=28, 51%)** | **Poor responders  (n=27, 49%)** | **Whole cohort**  **(n=55)** | **Good responders  (n=11, 37%)** | **Poor responders  (n=19, 63%)** | **Whole cohort**  **(n=30)** |
| **Age in years** | 17.5 (13.3-30.5) | 21.0 (15.0-54.0) | 20.0 (14.0-44.0) | 14.1 (11.1-22.3) | 15.0 (13.8-22.3) | 14.8 (13.1-23.2) |
| **Gender** |  |  |  |  |  |  |
| Male | 14 (50%) | 20 (74%) | 34 (62%) | 7 (64%) | 9 (47%) | 16 (53%) |
| Female | 14 (50%) | 7 (26%) | 21 (38%) | 4 (36%) | 10 (53%) | 14 (47%) |
| **Largest tumor diameter (cm)** | 9.4 (6.2-14.0) | 10.6 (8.1-14.2) | 10.0 (7.3-14.0) | 7.6 (7.2-10.3) | 8.3 (5.8-10.3) | 7.9 (6.5-10.5) |
| **Location** |  |  |  |  |  |  |
| Lower extremity | 23 (82%) | 17 (63%) | 40 (73%) | 11 (100%) | 16 (84%) | 27 (90%) |
| Upper extremity | 2 (7%) | 5 (18.5%) | 7 (13%) | 0 (0%) | 2 (11%) | 2 (7%) |
| Axial skeleton | 3 (11%) | 5 (18.5%) | 8 (14%) | 0 (0%) | 0 (0%) | 0 (0%) |
| Mandible | 0 (0%) | 0 (0%) | 0 (0%) | 0 (0%) | 1 (5%) | 1 (3%) |
| **Osteosarcoma subtype** |  |  |  |  |  |  |
| Conv. oteoblastic | 24 (86%) | 21 (78%) | 45 (82%) | 10 (91%) | 11 (58%) | 21 (70%) |
| Conv. chondroblastic^a^ | 0 (0%) | 2 (7%) | 2 (3%) | 0 (0%) | 7 (37%) | 7 (23%) |
| Telangiectatic | 4 (14%) | 2 (7%) | 6 (11%) | 1 (9%) | 1 (5%) | 2 (7%) |
| Periosteal | 0 (0%) | 1 (4%) | 1 (2%) | 0 (0%) | 0 (0%) | 0 (0%) |
| Extraskeletal | 0 (0%) | 1 (4%) | 1 (2%) | 0 (0%) | 0 (0%) | 0 (0%) |
| **Days 1^st^ DCE-MRI - start NAC** | 15.0 (8.0-21.0) | 15.0 (7.0-21.0) | 15.0 (8.0-21.0) | 12.0 (9.0-17.0) | 9.0 (6.0-17.0) | 10.0 (7.0-17.0) |
| **Days stop-NAC - 2^nd^ DCE-MRI**  **Days 2^nd^ DCE-MRI - resection** | 0.0 (0.0-0.0)  20 (13.0-24.8) | 0.0 (0.0-7.0)  15.0 (11.0-20.0) | 0.0 (0.0-5.0)  18.0 (13.0-21.0) | 8.0 (6.0-12.0)  8.0 (6.0-11.5) | 11.0 (7.0-14.5)  8.0 (6.5-13.0) | 9.0 (6.3-13.8)  8.0 (6.0-13.0) |
| **Treatment** |  |  |  |  |  |  |
| 2*MAP completed | 23 (82%) | 17 (63%) | 40 (73%) | 10 (91%) | 18 (95%) | 28 (93%) |
| 2*MAP not completed | 2 (7%) | 4 (15%) | 6 (11%) | 0 (0%) | 0 (0%) | 0 (0%) |
| AP cycles  Doxorubicin monotherapy | 3 (11%)  0 (0%) | 4 (15%)  2 (7%) | 7 (13%)  2 (3%) | 1 (9%)  0 (0%) | 1 (5%)  0 (0%) | 2 (7%)  0 (0%) |

**Supplemental Table 1 |** *Patient characteristics of the training and test cohorts. Patients were classified as good responders if pathological assessment showed good histological response after neoadjuvant chemotherapy, defined as <10% remaining viable tumor cells. If poor histological response was reported, defined as ≥10% remaining viable tumor cells after neoadjuvant chemotherapy, patient were classified as poor responders. Cohort characteristics are expressed in absolute numbers (%) or median (interquartile range). A = doxorubicin; cm = centimeter; conv. = conventional; DCE-MRI = dynamic contrast-enhanced MRI; M = methotrexate; n = number; NAC = neoadjuvant chemotherapy; P = cisplatin; T1w = T1-weighted. ^a^ Defined by >50% cartilaginous components in biopsy material.*
