# Supplementary material for: Evaluation of response to neoadjuvant chemotherapy in osteosarcoma using dynamic contrast-enhanced MRI: development and external validation of a model
Source: Skeletal Radiol. 2023 Jul 18;53(2):319–28. doi: 10.1007/s00256-023-04402-8 (PMC10730632; doi:10.1007/s00256-023-04402-8)
Supplement: Supplementary file 2 — ESM 2 (13.8 KB) [file 256_2023_4402_MOESM2_ESM.docx]

**Supplemental Material 2**

All training cohort patients were examined with a standard imaging protocol on a 1.5T (*n*=46) or 3T (*n*=9) MRI scanner (models: Intera (*n*=37), Ingenia (*n*=18); Philips Medical Systems). DCE-MRI was performed with a T1-weighted turbo field echo sequence and a temporal resolution of 1.0-2.0 seconds (TR/TE: 2.5-5.4/1.2-1.7ms, flip angle: 10-20°, FOV: 250-480x250-480mm^2^, acquisition matrix: 128-264x128-175, number of sections per time frame: 7-14 depending on tumor size, slice thickness: 5-16mm, acquisition time: 2.3-5.0min). Data acquisition and intravenous injection of gadolinium contrast medium (Dotarem, gadoteric, 0.5mmol/ml, Guerbet) with 2ml/s by an automatic injector were started simultaneously (followed by a saline flush).

Test cohort patients were examined with a standard imaging protocol on a 1.5T (*n*=22) or 3T (*n*=8) MRI scanner (models: PrismaFit (*n*=5), AvantoFit (*n*=4), Aera (*n*=2), Avanto (*n*=11), Symphony (*n*=3), SymphonyTim (*n*=2), TrioTim (*n*=3); Siemens). DCE-MRI was performed with a T1-weighted 2D Turbo Flash (*n*=19) or 3D Twist Vibe (*n*=11) sequence and a temporal resolution of 0.1-4.0 seconds: 2D Turbo Flash (TR/TE: 1100/2.9-4.1ms, inversion time: 560ms, echo train length: 1, flip angle: 12°, FOV: 192-256x192-256mm^2^, acquisition matrix: 96-256x96-256, number of sections per time frame: 1 (largest tumoral slice), slice thickness: 6-10mm, acquisition time: 0.7-3.3min, temporal resolution: 0.1-1.7s); 3D Twist Vibe (TR/TE: 3.9-5.2/1.7-2.5ms, echo train length: 2, flip angle: 9-12°, FOV: 106-259x141-259mm^2^, acquisition matrix: 92-192x92-192, number of sections per time frame: 7-19 depending on tumor size, slice thickness: 3.5-5.0mm, acquisition time: 1.5-2.8min, temporal resolution: 1.8-4.0s). Data acquisition and intravenous injection of gadolinium contrast medium (0.1mmol/kg) with 2ml/s by an automatic injector were started simultaneously (followed by a saline flush): Dotarem (gadoteric, 0.5mmol/ml, Guerbet, *n*=6; all 2D Turbo Flash), Gadovist (gadobutrol, 1mmol/ml, Bayer, *n*=11; *n*=1 2D Turbo Flash; *n*=10 3D Twist Vibe), Prohance (gadoteridol, 0.5mmol/ml, Bracco Imaging, *n*=1; all 3D Twist Vibe), Magnevist (gadopentetate, 0.5mmol/ml, Bayer, *n*=12; all 2D Turbo Flash).
